# Laboratory-Generated DNA Can Cause Anomalous Pathogen Diagnostic Test Results

**DOI:** 10.1128/Spectrum.00313-21

**Published:** 2021-09-15

**Authors:** Lindsey R. Robinson-McCarthy, Alexander J. Mijalis, Gabriel T. Filsinger, Helena de Puig, Nina M. Donghia, Thomas E. Schaus, Robert A. Rasmussen, Raphael Ferreira, Jeantine E. Lunshof, George Chao, Dmitry Ter-Ovanesyan, Oliver Dodd, Erkin Kuru, Adama M. Sesay, Joshua Rainbow, Andrew C. Pawlowski, Timothy M. Wannier, Nicolaas M. Angenent-Mari, Devora Najjar, Peng Yin, Donald E. Ingber, Jenny M. Tam, George M. Church

**Affiliations:** a Department of Genetics, Harvard Medical School, Boston, Massachusetts, USA; b Wyss Institute for Biologically Inspired Engineering, Harvard University, Boston, Massachusetts, USA; c Department of Systems Biology, Harvard Medical School, Boston, Massachusetts, USA; d Institute for Medical Engineering and Science, Massachusetts Institute of Technology (MIT), Cambridge, Massachusetts, USA; e Department of Global Health and Social Medicine, Harvard Center for Bioethics, Harvard Medical School, Boston, Massachusetts, USA; f European Research Institute for the Biology of Ageing, University Medical Center Groningen, University of Groningen, Groningen, The Netherlands; g Department of Electrical and Electronic Engineering, University of Bath, Bath, United Kingdom; h Department of Biological Engineering, Massachusetts Institute of Technology (MIT), Cambridge, Massachusetts, USA; i MIT Media Lab, Massachusetts Institute of Technology (MIT), Cambridge, Massachusetts, USA; j Vascular Biology Program, Department of Surgery, Boston Children’s Hospital and Harvard Medical School, Boston, Massachusetts, USA; k Harvard John A. Paulson School of Engineering and Applied Sciences, Harvard University, Cambridge, Massachusetts, USA; l Harvard-MIT Program in Health Sciences and Technology, Cambridge, Massachusetts, USA; m Blavatnik Institute, Harvard Medical School, Boston, Massachusetts, USA; University of Arizona/Banner Health

**Keywords:** COVID-19, nucleic acids, SARS-CoV-2, diagnostics

## Abstract

The coronavirus disease 2019 (COVID-19) pandemic has brought about the unprecedented expansion of highly sensitive molecular diagnostics as a primary infection control strategy. At the same time, many laboratories have shifted focus to severe acute respiratory syndrome coronavirus 2 (SARS-CoV-2) research and diagnostic development, leading to large-scale production of SARS-CoV-2 nucleic acids that can interfere with these tests. We have identified multiple instances, in independent laboratories, in which nucleic acids generated in research settings are suspected to have caused researchers to test positive for SARS-CoV-2 in surveillance testing. In some cases, the affected individuals did not work directly with these nucleic acids but were exposed *via* a contaminated surface or object. Though researchers have long been vigilant of DNA contaminants, the transfer of these contaminants to SARS-CoV-2 testing samples can result in anomalous test results. The impact of these incidents stretches into the public sphere, placing additional burdens on public health resources, placing affected researchers and their contacts in isolation and quarantine, removing them from the testing pool for 3 months, and carrying the potential to trigger shutdowns of classrooms and workplaces. We report our observations as a call for increased stewardship over nucleic acids with the potential to impact both the use and development of diagnostics.

**IMPORTANCE** To meet the challenges imposed by the COVID-19 pandemic, research laboratories shifted their focus and clinical diagnostic laboratories developed and utilized new assays. Nucleic acid-based testing became widespread and, for the first time, was used as a prophylactic measure. We report 15 cases of researchers at two institutes testing positive for SARS-CoV-2 on routine surveillance tests, in the absence of any symptoms or transmission. These researchers were likely contaminated with nonhazardous nucleic acids generated in the laboratory in the course of developing new SARS-CoV-2 diagnostics. These contaminating nucleic acids were persistent and widespread throughout the laboratory. We report these findings as a cautionary tale to those working with nucleic acids used in diagnostic testing and as a call for careful stewardship of diagnostically relevant molecules. Our conclusions are especially relevant as at-home COVID-19 testing gains traction in the marketplace and these amplicons may impact on the general public.

## INTRODUCTION

The coronavirus disease 2019 (COVID-19) pandemic has brought about an unprecedented need for population-scale pathogen testing. Nucleic acid-based tests, particularly reverse transcription-quantitative PCR (RT-qPCR) tests, are the most frequently used and are currently the gold standard for severe acute respiratory syndrome coronavirus 2 (SARS-CoV-2) diagnostics (1). RT-qPCR tests are highly sensitive and specific (2). Stringent controls are used in diagnostic laboratories, including engineering and molecular controls, as well as proper laboratory practices and personal protective equipment, to prevent sample contamination and false-positive results. These controls are not typically focused on preventing contamination of the laboratory worker with nonhazardous material.

The pandemic has also led many research and clinical diagnostic laboratories to change focus to SARS-CoV-2. Many laboratories now routinely work with SARS-CoV-2 nucleic acids in the course of diagnostic development and basic biological research. Although laboratory workers stringently follow standard laboratory practices while working with these noninfectious and nonhazardous nucleic acids, researchers can still inadvertently contaminate laboratory surfaces and equipment with these nucleic acids, and DNA is exceptionally stable in these environments. Once contaminated, complete removal of DNA from these spaces can be difficult (3), which has typically been an issue that affects sample quality and experiments.

Here, we present the cases of 15 researchers who received positive SARS-CoV-2 tests through routine surveillance testing at their institutions. We show that these were likely anomalous positive tests caused by contamination by laboratory-generated SARS-CoV-2 DNA. We find widespread and persistent laboratory contamination with amplified SARS-CoV-2 DNA. We identified this contamination and anomalous positive test results in multiple laboratories across two institutions. Given the prevalence of SARS-CoV-2 research, these are likely not isolated incidents.

Testing capacity in the United States has expanded from thousands of tests to currently 1 to 2 million laboratory-based tests per day (4). Public health experts estimate that we need approximately 9 million tests per day to accurately determine the extent of SARS-CoV-2 prevalence in the population (5). Surveillance testing of asymptomatic populations, such as has been pioneered by universities nationwide, is needed to reach this goal, and anomalous positive test results are likely to scale with testing. Following CDC guidelines, individuals with positive tests are removed from the testing pool for 90 days, leaving those with anomalous positive tests susceptible to infection that may go undetected (6). While these anomalous positive tests constitute a very small number relative to the total number of tests performed at these institutions, these cases serve as a powerful reminder that noninfectious material can still result in significant public health implications.

## RESULTS

### A case study in SARS-CoV-2 nucleic acid researcher contamination.

**FIG 1:**
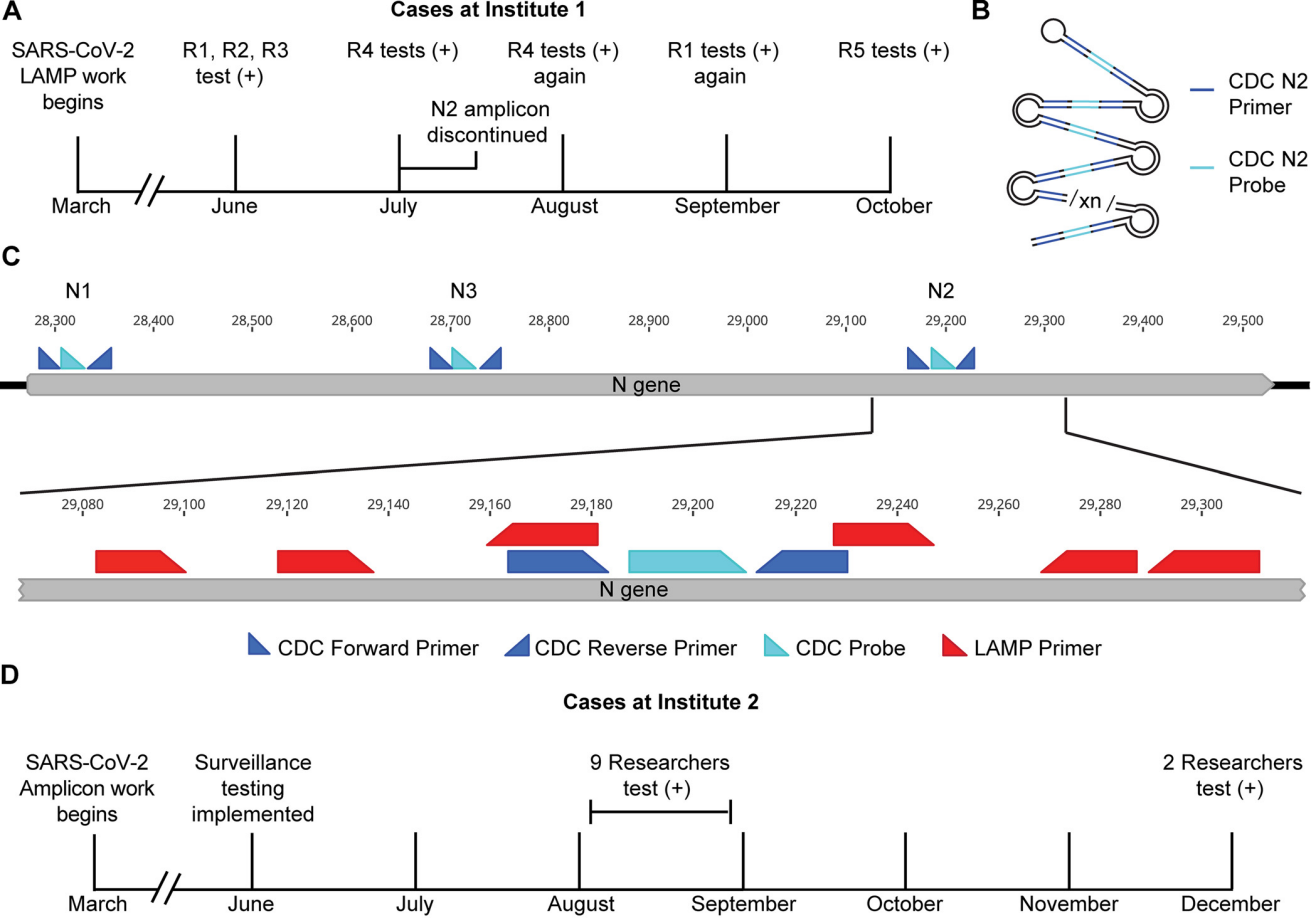
Positive SARS-CoV-2 tests among researchers at two institutes. (A) Timeline of positive tests at institute 1. R1, R2, R3, R4, and R5, researchers 1 to 5. (B) Schematic of the LAMP product produced in this laboratory highlighting the diagnostic RT-qPCR primer/probe annealing sites. (C) Schematic of SARS-CoV-2 N gene with diagnostic and research (LAMP) primer binding sites annotated. (D) Timeline of positive tests at institute 2. One individual tested positive in the initial group in August and again in December, giving a total of 10 individuals with positive tests.

Following a state-imposed stay at home order, a back-to-work plan for a research institute called for community-wide SARS-CoV-2 surveillance testing using self-administered nasal swabs. This testing program identified an epidemiologically linked cluster of SARS-CoV-2-positive researchers in one laboratory. Additional positive tests from researchers in this group were reported in the 3 months following the initial results. In total, five members of this group tested positive, with two individuals testing positive on two separate occasions (Fig. 1A). In all cases, university and state health departments were notified. In accordance with state and CDC guidelines, these researchers and their close contacts completed 10- to 14-day isolation periods without further incident.

When examining these incidents in detail, several aspects were inconsistent with SARS-CoV-2 infection and epidemiology. In all cases, the affected researchers and their contacts did not present with any clinical manifestations of SARS-CoV-2 infection. In the months that these positive tests occurred, the state of Massachusetts reported between 0.87 and 22 cases per 100,000 residents per day (7). Given an estimated rate of completely asymptomatic infections of between 20 and 33% (8, 9), we would expect fewer than one asymptomatic infection in this laboratory of approximately 50 people over this time period. All close contacts of affected researchers tested negative. Subsequent or follow-up tests of affected researchers by other diagnostic laboratories were negative. We were not able to obtain serological testing of these researchers to confirm whether they were exposed to SARS-CoV-2. We investigated these anomalous test results.

The researchers all worked in a laboratory that was developing SARS-CoV-2 molecular diagnostics, which involved investigating loop-mediated isothermal amplification (LAMP) of reverse-transcribed, noninfectious, and nonhazardous SARS-CoV-2 sequences. This laboratory also did not work with infectious virus or patient samples. The target sequence included the CDC “N2” locus that is now widely used in RT-qPCR diagnostics (Fig. 1B and C) (10). It was also the sole viral locus used in the initial community surveillance tests that were administered to the researchers. The diagnostic laboratories that produced the negative follow-up testing results employed molecular tests that detect the N1 and N3 loci.

We later learned that anomalous SARS-CoV-2 test results among researchers were not limited to a single laboratory. At a second research institute, 10 researchers in three separate research groups tested positive for SARS-CoV-2, with one testing positive on two separate occasions (Fig. 1D). These researchers shared common spaces and equipment, including thermocyclers, benches, and centrifuges. For all researchers, follow-up testing yielded no confirmation of infection. Similarly, none of the researchers or their close contacts exhibited symptoms of COVID-19.

### Identifying the source of laboratory DNA contamination.

**FIG 2:**
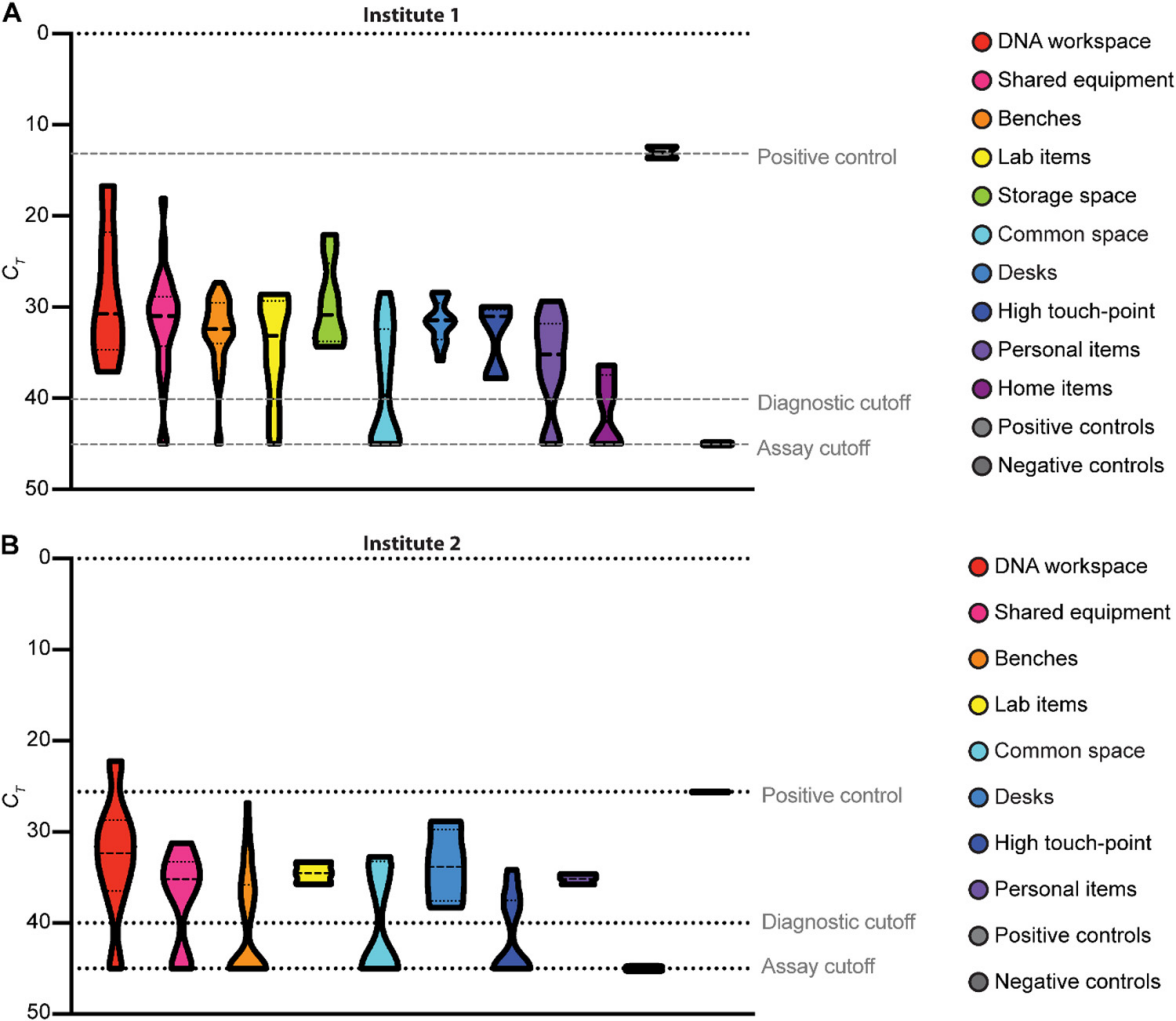
Laboratory contamination with SARS-CoV-2 amplicons. Surfaces and equipment throughout the laboratory space were sampled with dry cotton swabs. Swabs were eluted in TE buffer, and the eluate was analyzed using a qPCR assay with the CDC N2 primer/probe. (A) Laboratory contamination at institute 1. (B) Laboratory contamination at institute 2. *C_T_*, cycle threshold.

To determine whether amplified DNA products that could affect test results were widely present in the laboratory, we collected swabs from various surfaces and equipment throughout the laboratory, eluted any captured DNA, and performed qPCR using the CDC N2 primer/probe set. The qPCR did not include reverse transcriptase and therefore only detected amplified cDNA, not viral RNA. Although the researchers had followed standard practices for working with amplified nucleic acids, including physical separation of pre- and postamplification workspaces, we found that nearly every surface had detectable quantities of N2 amplicon (Fig. 2A). The highest levels of DNA were found in the workspaces used for SARS-CoV-2 LAMP reactions, on shared equipment used for analyzing these products and the refrigerators and freezers used to store these products. Common areas, sinks, and door handles in the laboratory were also positive. Contaminated surfaces were identified in four separate rooms. Amplicons were also identified on researchers’ personal items and had spread into the home of at least one researcher. A contaminated doormat deposited amplicons onto the shoes of a researcher’s spouse who was never physically present in the laboratory.

Sampling of the laboratory space in the second research institute revealed that there was also widespread N2 amplicon contamination in the space (Fig. 2B), which was likely generated during the course of development of SARS-CoV-2 diagnostics. This contamination was found in multiple rooms across two floors of the building.

### Laboratory DNA contamination is persistent.

**FIG 3:**
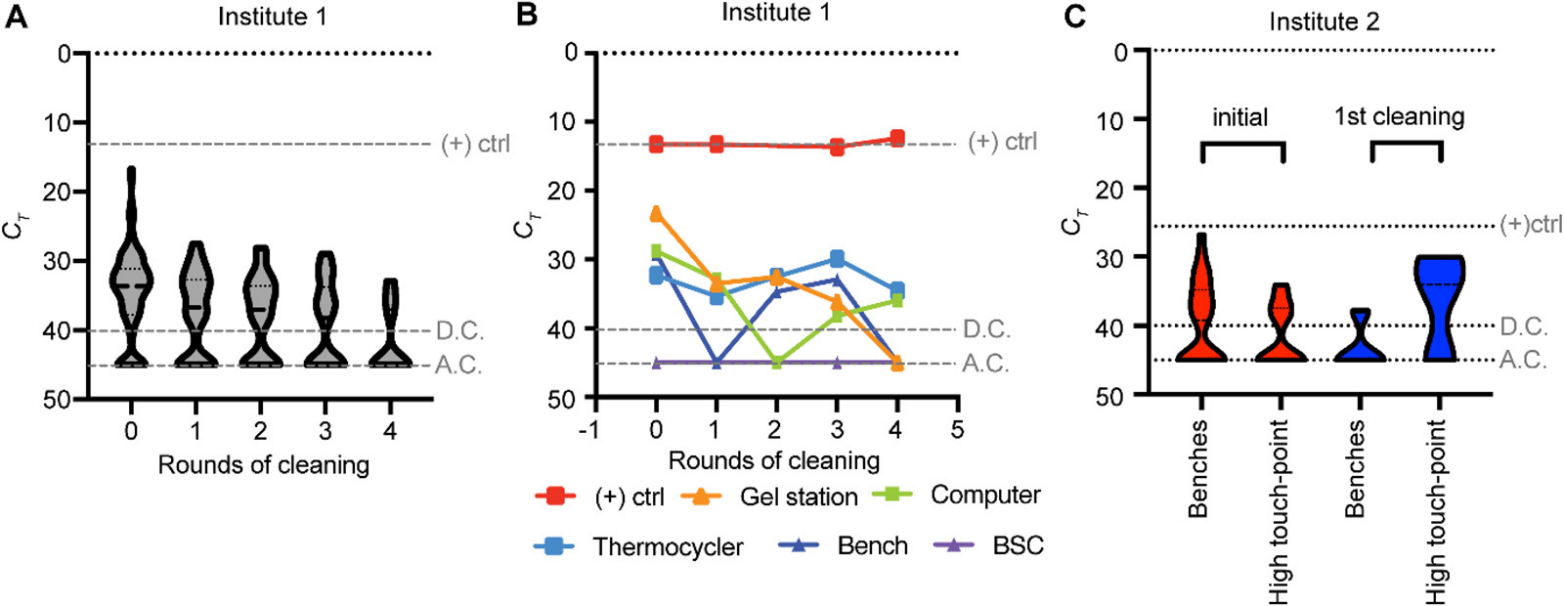
SARS-CoV-2 DNA remaining after cleaning. (A) Swabs were collected before and after multiple rounds of cleaning at institute 1. Data shown are for all surfaces tested. 0, before cleaning. (B) Select surfaces from institute 1 over successive rounds of cleaning. BSC, biosafety cabinet. (C) Swabs were collected before and after one round of cleaning for benches and high-touch-point areas at institute 2. (+)ctrl, positive control; D.C., diagnostic cutoff; A.C., assay cutoff.

Following the discovery of extensive laboratory contamination, work using the N2 amplicon in the first laboratory ceased. Repeated efforts to decontaminate the laboratory were met with limited success. We performed iterative cycles of cleaning, swabbing, and qPCR detection of remaining DNA. These efforts relied on chemical agents, including bleach, DNA Away, and hydrogen peroxide solutions. After five rounds, we were able to reduce, but not eliminate, the LAMP DNA (Fig. 3A). Some individual surfaces remained free of detectable amplicons throughout our survey period, some remained contaminated, and some remained clean after the first rounds of decontamination (Fig. 3B). Smooth surfaces like bench tops, fume hoods, and biosafety cabinets were the easiest to decontaminate, while those with grooves, such as thermocyclers and pipettes, were more resistant to decontamination. However, some surfaces and equipment oscillated between negative and positive. This likely represents transfer of amplicons from an unknown contamination source, or it may represent the stochastic nature of our sampling. Either scenario highlights that complete removal of contaminating DNA may not be possible. Indeed, three of the positive test results occurred after these widespread cleaning procedures were implemented. Similar results were observed at institute 2. After cleaning the affected surfaces with 5% bleach, the levels of amplicon contamination decreased on laboratory benches but increased on high-touch-point areas, likely due to transfer from other contaminated areas (Fig. 3C).

## DISCUSSION

The SARS-CoV-2 pandemic has brought about an era during which, for the first time in history, humans are subject to recurrent, population-scale nucleic acid testing. It has also prompted laboratories around the world to research SARS-CoV-2 biology and improved nucleic acid diagnostics. Therefore, nucleic acids with the capacity to trigger positive diagnostic test results are likely present at nearly every research institute and many clinical laboratories. We have presented instances in which positive SARS-CoV-2 tests were likely triggered by laboratory-generated DNA, and we identified widespread laboratory DNA contamination. Anomalous tests among researchers have been reported at other research institutes as well, with one report confirming lack of infection through negative serology tests (11, 12). While the cases reported here occurred in research settings, this is an issue that can confound clinical and clinical diagnostic laboratories, as surface and personnel contamination with both nonhazardous and infectious agents can readily occur in these settings as well (13). These cases are examples of what has the potential to be a widespread problem in research and diagnostic laboratories.

The rapid spread and persistence of contaminating DNA throughout a laboratory and beyond raises important issues regarding the stewardship of nucleic acids that can confound test results. The contamination in these laboratories was likely exacerbated by the use of LAMP reactions, which generate concatenated copies of the amplified target sequence (14). A single molecule of LAMP product captured in a diagnostic assay is likely sufficient to produce a positive RT-qPCR result. As a contaminant, LAMP products are more likely to persist in the environment due to their high molecular weight and structure. Additionally, the presence of multiple target sequences per molecule makes LAMP products more resistant to nuclease activity that would render them undetectable in diagnostic tests. Highly structured loops may also make these products more resistant to degradation than linear or circular DNA products. While LAMP products may be particularly problematic in this regard, DNA that can confound SARS-CoV-2 tests can come from many sources, including amplified sequences and plasmid DNA.

The widespread presence of amplified SARS-CoV-2 DNA products in both research institutes had impacts that extended beyond the research teams that generated the amplicons. The CDC currently recommends that individuals who test positive not receive additional diagnostic tests for 90 days, given the long residence time of residual SARS-CoV-2 RNA in infected patients (6). A subsequent true infection could go undetected and spread to others. Several of the researchers who tested positive were not involved in SARS-CoV-2 research. They likely unknowingly interacted with contaminated equipment or surfaces. It is possible that other researchers, janitorial, security, and maintenance staff, and those involved in the disposal of laboratory waste were all unknowingly exposed. It also highlights the possibility of contaminating DNA spreading to public spaces outside the laboratory. In the case of the researcher’s home, their spouse had never entered the laboratory and had personal items that were positive for SARS-CoV-2 amplicons. These anomalous positive tests can also trigger unnecessary infection control procedures, which can tax institutional, hospital, and public health resources.

Laboratory-generated nucleic acids have the potential to affect the community beyond those directly associated with the laboratory. Wastewater surveillance has been implemented as an early warning system to detect community spread that has fallen outside the framework of individual testing (15, 16). Similar or more widespread measures may be implemented for other pathogens in the future. Increased use of viral DNA constructs in laboratories could produce false positives in wastewater samples as well, especially in communities that have a high concentration of academic and industrial research laboratories. Research and diagnostic laboratories have the opportunity to design our processes to preserve the integrity of the diagnostic tests, while ensuring that important work focused on responding to this crisis continues unabated.

In order to reduce the risks involved with working with nucleic acids that can compromise testing, we have devised a series of common-sense recommendations for laboratory workers. These nucleic acids include not only amplicons from LAMP, recombinase polymerase amplification (RPA), PCR, or other methods but also plasmids and any other nucleic acid-containing SARS-CoV-2 sequences. Steps to prevent laboratory contamination, such as engineering controls, good work practices, and proper personal protective equipment (PPE), should be coordinated in advance of the initiation of research. In the cases outlined here, the researchers used basic engineering controls of separating pre- and postamplification workspaces. More stringent controls would include physical separation of these spaces in different rooms and unidirectional workflow from pre- to postamplification spaces. The affected researchers wore lab coats, gloves, and surgical masks. Unidirectional workflow would also ensure that PPE worn in postamplification spaces was not worn in other laboratory or clinical spaces. These controls would all reduce the likelihood of contaminating personal items belonging to researchers and technicians and are indeed standard practice in many clinical diagnostic laboratories. Periodic environmental surveys of laboratory surfaces and personal items reduced the frequency of anomalous positive test in the affected laboratories and should be included in experimental/diagnostic design.

A plan for how to respond to a presumptive positive test and how to verify it should be in place before initiation of work with these nucleic acids. When designing experiments, attempts should be made to limit the likelihood that a contaminant in the laboratory would interfere with the result for every single approved test. In the research laboratory described here, it was possible to follow up on the initial positive result using another test provider that used the N1 and N3 primer-probe sets.

Whenever feasible, unique nucleotide substitutions, or “watermarks,” should be introduced to distinguish laboratory products from circulating pathogens. These watermarks would ideally prevent detection by diagnostic tests (e.g., nucleotide substitutions in qPCR primer or probe annealing sites). Watermarks have been used in engineered microorganisms to differentiate recombinant viruses from circulating viruses (17, 18). Whenever possible, controls that prevent carryover contamination, such as the dUTP/uracil *N*-glycosylase (UNG) system, should also be used (19). This system incorporated uracil into the final amplified product. UNG is added to the initial reaction mixture to degrade any amplified product that may have been carried over from previous tests and/or surface contamination.

Laboratories should declare and post notices if they produce problematic nucleic acids and indicate their presence to those who handle waste streams. This would help direct those with positive test results to the resources for verification. Rigorous engineering controls and standard operating procedures for working with these DNA products should be in place, including proper use of fume hoods or biosafety cabinets when working with amplified nucleic acids (20). These procedures are typically focused on maintaining sample purity; now, attention must be paid to the handling of amplified DNA to prevent contamination of the researcher and environment. Special care should be taken to treat waste before disposal to prevent contamination of the environment and of those handling the waste. Laboratory hygiene is critical: testing surfaces, tracing sources of contamination, and cleaning of equipment and surfaces in this laboratory with bleach has reduced the likelihood that additional laboratory personnel will test positive for SARS-CoV-2. Finally, for DNA species that can interfere with testing, the traditional view of laboratory contaminants needs to change. These are no longer merely a problem of contaminated experiments. They can put in question one’s health status, cause unnecessary isolations and quarantines, impose significant stress, impact businesses and schools, and skew wastewater or similar sentinel testing programs (21).

The advent of at-home testing poses another challenge in preventing environmental contamination with interfering DNA. The first completely at-home test to receive emergency use authorization from the Food and Drug Administration is a LAMP-based test (22). It is important that these tests are developed with the issue of contaminating DNA in mind, to prevent as much as possible the release of SARS-CoV-2 DNA in the home and environment.

PCR-based testing is the current gold standard for SARS-CoV-2 testing, and these tests are highly sensitive and accurate. Even in these reported instances, the tests are performing as intended to detect SARS-CoV-2 sequences. We emphasize that research-produced nucleic acids triggering a SARS-CoV-2-positive diagnostic test result is a rare circumstance. For those engaged in research that generates nucleic acids with the capacity to interfere with testing or surveillance, we have a responsibility to not contaminate our environments in ways that will undermine trust and impede life-saving public health initiatives, for SARS-CoV-2 or otherwise.

## MATERIALS AND METHODS

### Researcher test results.

Test results from individual researchers were volunteered and directly communicated. The Institutional Review Board of the Harvard Faculty of Medicine determined that this did not constitute research on human subjects.

### Laboratory sampling for DNA amplicon contamination.

Laboratory surfaces were swabbed for contamination using clean Q-tips brand cotton swabs. Swabs were prepared for sampling in a clean biosafety cabinet that had never been used for SARS-CoV-2 work. First, the working area and equipment were cleaned with a solution of either 2% bleach or 1% hydrogen peroxide, followed by 70% ethanol. Swabs were cut to fit inside the tubes, which were closed and placed in a clean box. Gloves were worn while sampling and changed between samplings. To sample a surface, a clean swab was removed from its tube, rubbed over the surface to be tested, and placed back in the tube. Two swabs were left unopened to serve as negative controls. Once swabbing was complete, 300 μl of TE buffer (10 mM Tris-HCl, 0.1 mM EDTA, pH 8.0) (catalog number 12090015; Invitrogen) was added to each tube. The tubes were then incubated at 37°C for 30 min, vortexed briefly, and then centrifuged at high speed for 30 s. In a biosafety cabinet, 50 μl of the swab eluate was transferred from each tube to a corresponding well of a sterile 96-well PCR plate, sealed, and stored at −20°C until analysis.

### Analysis of swab eluate by qPCR.

The qPCR mixture with primers and probe was prepared for analysis of the CDC N2 amplicon, using N2 primer and probe (2019-nCov CDC EUA kit, product number 10006770; Integrated DNA Technologies) and Luna universal probe qPCR master mix (product number M3004; New England Biosystems). To analyze 96 samples, including controls, 300 μl of water and 200 μl N2 of primer/probe mixture were added to a tube containing 1 ml of 2× Luna probe mixture. A 96-well qPCR plate was placed on a cold block on ice, and 15 μl of this mixture was added to each well. Using a multichannel pipette, 5 μl of each swab eluate was added into a corresponding well of the plate containing the qPCR mix. On each plate, negative-control swabs, wells containing only TE buffer, and positive controls known to contain the N2 DNA amplicon were also analyzed. The plate was sealed with optical film and placed in a LightCycler 96 qPCR machine (Roche), which was run for 45 cycles. Data were analyzed using Prism 8 software (GraphPad). Values for samples with undetectable DNA (no cycle threshold [*C_T_*] value) were set at a *C_T_* of 45 for purposes of visualization.
